# A blended framework for audio spoof detection with sequential models and bags of auditory bites

**DOI:** 10.1038/s41598-024-71026-w

**Published:** 2024-08-30

**Authors:** Misaj Sharafudeen, Vinod Chandra S S, Andrew J., Yuichi Sei

**Affiliations:** 1 Machine Intelligence Research Laboratory, Department of Computer Science, University of Kerala, Thiruvananthapuram, Kerala India; 2 Department of Computer Science and Engineering, Manipal Institute of Technology, Manipal Academy of Higher Education, Manipal, 576104 Karnataka India; 3 Graduate School of Informatics and Engineering, The University of Electro-Communications, Tokyo, Japan

**Keywords:** Computational biology and bioinformatics, Machine learning

## Abstract

An automated speaker verification system uses the process of speech recognition to verify the identity of a user and block illicit access. Logical access attacks are efforts to obtain access to a system by tampering with its algorithms or data, or by circumventing security mechanisms. DeepFake attacks are a form of logical access threats that employs artificial intelligence to produce highly realistic audio clips of human voice, that may be used to circumvent vocal authentication systems. This paper presents a framework for the detection of Logical Access and DeepFake audio spoofings by integrating audio file components and time-frequency representation spectrograms into a lower-dimensional space using sequential prediction models. Bidirectional-LSTM trained on the bonafide class generates significant one-dimensional features for both classes. The feature set is then standardized to a fixed set using a novel Bags of Auditory Bites (BoAB) feature standardizing algorithm. The Extreme Learning Machine maps the feature space to predictions that differentiate between genuine and spoofed speeches. The framework is evaluated using the ASVspoof 2021 dataset, a comprehensive collection of audio recordings designed for evaluating the strength of speaker verification systems against spoofing attacks. It achieves favorable results on synthesized DeepFake attacks with an Equal Error Rate (EER) of 1.18% in the most optimal setting. Logical Access attacks were more challenging to detect at an EER of 12.22%. Compared to the state-of-the-arts in the ASVspoof2021 dataset, the proposed method notably improves EER for DeepFake attacks by an improvement rate of 95.16%.

## Introduction

Voice biometrics is a viable option among the various biometric techniques, such as facial, iris, and fingerprint recognition^1^. It is also considered more economical and computationally efficient than other biometric systems^2^. Automatic speaker verification (ASV) is commonly incorporated into an array of devices, such as smartphones and smart speakers, for user authentication. It is used in different sectors, such as banking, e-commerce systems, home automation, and app login^3^. For example, our voice can be used to command devices such as Apple Siri, Lenovo ASV, or Google Home to open/close doors, set reminders, make phone calls and texts, unlock cellphones, or even play music. Citibank was the first bank to implement voice biometrics authentication for phone banking customers, allowing them to use their voice prints for authentication^4^. In addition, voice is a secure and convenient way to identify the client regarding mobile banking, both at the start of a transaction and during the instruction for trade or payment. This could add an extra layer of security to mobile banking^5^.

The goal of this study is to devise a reliable approach for accurately detecting various forms of voice spoofing attacks, such as logical access attacks (Voice Conversion (VC) and Text-To-Speech (TTS) synthesis) and deepfake attacks (VC and TTS without speaker verification). It also aims to detect physical access attacks (mimicry, twins, replay) that can be used to fool ASV systems. Deepfake incidents have become a prevalent form of attack, notably in images and videos, with the surge in generative AI tools^6^. These attacks can be difficult to detect using audio cues found in the recordings^7^. To address this challenge, we develop a countermeasure for audio spoofing in the ASV system by classifying entirely spoofed audio in an efficient learning method from bags of auditory cues. Audio sequences are varied in length, as are the features extracted collectively from each timestamp. They must be restructured to a fixed number of descriptors to ensure an accurate and efficient detection system. This research aims to provide a solution that strives for a minimal error rate.

**Figure 1:**
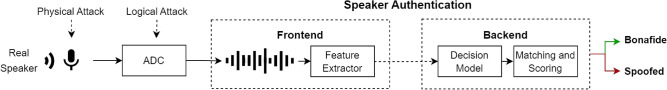
Framework of an automated speaker verification system.

Audio biometrics and automatic speaker verification systems ensure that only authenticated speech is accepted^8^. The ASV system examines a voice input received through a microphone and decides whether to accept or decline the claimed identity. Speaker verification is used to assess if the speech uttered by an individual is authentic. For effective operation, both front-end and back-end components are essential. As depicted in Fig. 1, the front end processes the input speech signal and conducts a validity check. It performs speaker verification by contrasting the voice of the speaker to the speech of authorized users stored in the system’s database. This involves gathering speaker data and voice signal authenticity details through characteristics like phase, time delay, frequency, and pitch. Feature extraction analyzes these characteristics using short-term power spectrum features, short-term phase features, and long-term processing steps. Frequency domain feature extraction techniques are commonly used for accuracy in predicting the human auditory and vocal tracts. The back-end classification model, employing deep learning or machine learning algorithms, is trained on a robust speech dataset. This dataset requires audio files, speaker information, and audio and speaker-specific metadata for effective speaker verification in diverse acoustic environments^7^. The ASV system, tested on a standardized audio dataset, delivers accurate acceptance or rejection of incoming data.

The speaker verification process involves analysing a claimed speaker voice to determine if the claim is authentic. The analogue speech signal is converted to digital through a sampler, quantiser, and encoder to extract the relevant features. Feature extraction methods such as Linear Predictive Coding (LPC)^9^, Mel Frequency Cepstrum Coefficients (MFCC)^10^, and Linear Predictive Cepstrum Coefficients (LPCC)^11^ are used to extract the necessary features. Backend of the system involves a classification model that assesses the speech characteristics of the test data in comparison to those of the user, determining if the claim is valid.

It is possible to generate fake voice recordings by altering the original audio signal through recording, manipulation, or imitation. Logical Access (LA) attacks include voice conversion, whereas DeepFakes (DF) include speech synthesis. A synthetic voice that sounds similar to the already registered speaker voice is created with voice conversion. Furthermore, speech synthesis involves generating artificial speech that imitates the target speaker. Speech synthesis attacks are more advanced and involve collecting voice samples of the victim, utilising machine learning techniques to train the voice biometric models, and creating new voice attack samples^12^. Free tools such as Google Wavenet and Tacotron are available for training voice models^13^. PA attacks include Mimicry, Twins, and Replay spoofing. In these cases, a fraudster records the voice of a registered speaker and plays it for the ASV system to gain access.

ASV spoof detection systems continually evolve to bypass existing security measures, necessitating the development of more advanced detection methods that can adapt to and counteract these evolving threats. These systems rely heavily on analyzing various audio signal features and their attributes to distinguish between genuine and spoofed audio. By incorporating detailed insights into the characteristics of audio signals, such as spectrograms that visualize frequency changes over time, researchers can enhance the effectiveness of spoof detection algorithms. Features extracted from raw audio waveforms further contribute to this goal. Existing models have traditionally focused on sequential signal processing methods, but utilizing structured pattern analysis techniques with large datasets can significantly improve system performance. Effectively identifying different types of spoofing attacks, such as impersonation or playback attacks, relies on extracting subtle audio cues from legitimate user recordings. These cues are essential within a speaker verification system. The traces of synthesis in spoofed waveform features are so subtle that detecting ASV spoof attacks becomes nearly impossible^14^.

Our goal is to create a robust method that can identify logical, and deepfake spoofing, without relying on knowing which technique was used. We used datasets from the latest challenge ASVspoof2021^15^, emphasising the detection of logical and deepfake attacks.

The key contributions of the paper can be summarized as follows:

- The study detects logical access and deepfake attacks by integrating raw audio file components and time-frequency representation spectrograms into a lower-dimensional space using sequential prediction models.
- Our methodology finds a representation that signifies the relevance of each timestamp by predicting the amplitude of the following timestamp and clustering the time-width feature vector into Bags of Auditory Bites (BoAB).
- The BoAB technique regularizes the number of features to a defined set of values by compressing and bounding dimensionality to a finite count and transforming vocabulary of auditory words of the train set into codewords.
- We discovered that logical access attacks are more difficult to detect due to voice conversion algorithms, which use voice samples as input rather than deepfakes.

### Related works

Automated Speaker Verification and spoofing detection experts have devoted more effort to generating tough acoustic features for detecting voice spoofing attacks. Current research has looked into many audio characteristics, such as phase spectrum, magnitude, pitch, spectral centroid, etc., to differentiate between true and faked human speech^16^. Additionally, Gaussian Mixture Models (GMM) and Support Vector Machine (SVM) classifiers have been explored in previous studies for voice spoofing recognition^17,18^. The studies available in existing literature conducted in the datasets obtained from ASVspoof challenges from 2015 to 2021 are briefed.

Extracting significant and desired descriptors from raw audio signals and interpreting them can be challenging due to their varied waveform size. A spoof-print system using linear discriminant analysis to transform feature vectors into a lower-dimensional vector space was proposed^19^. Cosine similarity computed between the learned and incoming vectors determined whether the speaker is genuine, with an error rate of 5.55% when tested on the ASVspoof2019 dataset. Yaguchi et al. used a combination of spatial and spectral data for a 7% EER Replay Attack Detection (RAD) technique^20^. Ren et al. proposed a Deep Learning based RAD (DL-RAD) scheme to discover voiced audio segments and achieved an error rate of 4.03^21^. In^22^, the authors presented a method for detecting replay attacks using a weighted linear combination of classifier scores achieving lower equal error rates of 0.3% and 4.8%. The study in^23^ introduces a device-related linear transformation approach that effectively detects replay speech and outperforms other features, highlighting the importance of device information in replay attacks. Zhang et al.^24^ introduced an improved countermeasure for automatic speaker verification systems to detect short-generated spoofed speech segments at finer temporal resolutions enabling simultaneous segment and utterance level detection.

Studies integrate temporal and frequency domain descriptors to aim for better predictions. Previous studies heavily relied on a single audio descriptor, usually from spectrograms, potentially inadequate for detecting sparse spoofing traces^25^. Zhang et al.^26^ showcased significant EER reduction by integrating a segment-based word CQCC and average zero-cross rate technique. The dependencies between succeeding audio bits could alone not generalise to unseen spoofing assaults but were observed to resist logical attacks. In^27^, a method separates high and low-frequency elements into audio frames. It employs local statistics to collect information about the user voice tract and integrating multiple domain features could add to the efficiency of prediction. Speech processing using neural networks uses deep learning to remove noise from speech signals, enhancing their quality, pattern learning and categorisation^16^. The study by Zhou et al. proposes an anti-spoofing measure in two stages: feature extraction in the form of Mel-Frequency Cepstral Coefficients (MFCC) and Gammatone Cepstrum Coefficient (GTCC) from audio signals, and classification, where a Bi-directional Long Short-Term Memory Network was employed^28^. Data augmentation techniques were combined with hybrid feature extraction (MFCC+GTCC) and LSTM back-end in detecting deepfakes in an ASV system^29^.

Recent insights have observed the effectiveness of using Time Delay Neural Networks (TDNN) for automatic speaker verification and anti-spoofing^30,31^. The model used a multi-level feature aggregation block and a channel and context-dependent statistics pooling block, as well as varying the number of filters of convolutional layers^32^. The model achieved an error rate of 4.47% on the ASVspoof2019 dataset. The proposed DBiLSTM model in^33^ consisting of 10 Bi-LSTM layers, 64 hidden units, an FC layer and a SoftMax layer achieved an Equal Error Rate (EER) of 0.74, which is the least error rate among all the compared papers. Xie et al.^34^ proposed Temporal Deepfake Location, a fine-grained method for detecting partially spoofed audio by utilizing an embedding similarity module and temporal convolution operation to enhance frame-level authenticity identification, achieving an EER of 11.23 on the DF track. Luo et al. in^35^ propose a capsule network for enhanced generalization in detecting audio spoofing attacks, improving upon the limitations of current convolutional neural network-based methods. Regional energy features in^36^, were experimentally proven to be more effective and computationally efficient than traditional frame-based energy features, achieving significant improvements across multiple datasets including ASVspoof 2019 and 2021. A deep correlation network (DCN) significantly improved detection performance by learning and utilizing common information between different audio features^37^. This shows the potential of deep learning models for audio scam detection.

Integrating relevant temporal and spectral descriptors into a deep neural framework could effectively build an accurate ASV system. Previous literature has mostly focused on blind predictions using sequential models, and there has been little study of the structure of feature descriptors. A more intrinsic structured pattern learning from large amounts of data could develop the system.

## Materials and method

This research combines audio file features extracted from raw audio files, and time-frequency converted spectrograms that are interpolated to a lower dimension space using sequential models. This feature set of varied sizes is further regularised by clustering using the feature standardising procedure named Bags of Auditory Bites (BoAB). The transformed feature space is then mapped to predictions using a machine learning algorithm to classify between bonafide and spoofed speeches. The proposed architecture was then trained and tested on multiple public datasets to authenticate effectiveness of the model.

### Dataset

We chose the Logical Access (LA) and DeepFake (DF) dataparts of the well known ASVspoof2021 challenge datasets^15^. The ASVspoof2021 challenge is a community-led initiative to promote awareness of spoofing and to develop countermeasures. It involves three different tasks aimed at creating tools to detect genuine speech from spoofed speech, utilizing the biggest open-access dataset available for evaluating anti-spoofing countermeasures. The LA evaluation data consists of a collection of genuine and synthetic utterances that were transmitted across Voice over Internet Protocol (VoIP) and Public Switched Telephone Network (PSTN), which can be subject to encoding and transmission artifacts. These artifacts may be present in the recordings and can affect the accuracy of a system to detect spoofing. The DF detection task is designed to identify artificial audio generated by combining natural and artificially created utterances from Text-to-Speech and Voice Conversion technologies. It is comparable to the LA task, although it does not include Speaker Verification during the generation process. The DF evaluation data is a mix of real and falsified audio recordings that have gone through a range of popularly used media storage formats. The ASVspoof 2019 LA assessment data and other sources have been used to create the 2021 DF evaluation data which includes spoofing attacks generated by spoofing algorithms. The database contains a total of 181567 recordings in the LA data part, and 611830 recordings in the DF detection data, including genuine recordings from 1,066 speakers. The logical and deepfake attacks include 13 synthetic speech or voice conversion attacks, 2 microphone types (far-field and close-talking), 3 recording environments (studio, home, and telephone), and 8 attack scenarios (3 speaker-dependent, 2 speaker-independent, and 3 multi-speaker). Additionally, there are 4 difficulty levels (Easy, Medium, Hard, and Extreme) and 4 types of data (development, evaluation, blind, and unseen).

We balanced the dataset to ensure uniformity in training. Hence, we selected 18,452 audio waveforms each from the bonafide and spoofed categories of the LA data, and 5535 waveforms each from the bonafide and spoofed classes of the DF data. The total data was highly imbalanced, with only 11.31% belonging to the LA spoofed set and 3.75% belonging to the DF spoofed set.

### Audio spoof detection framework

**Figure 2:**
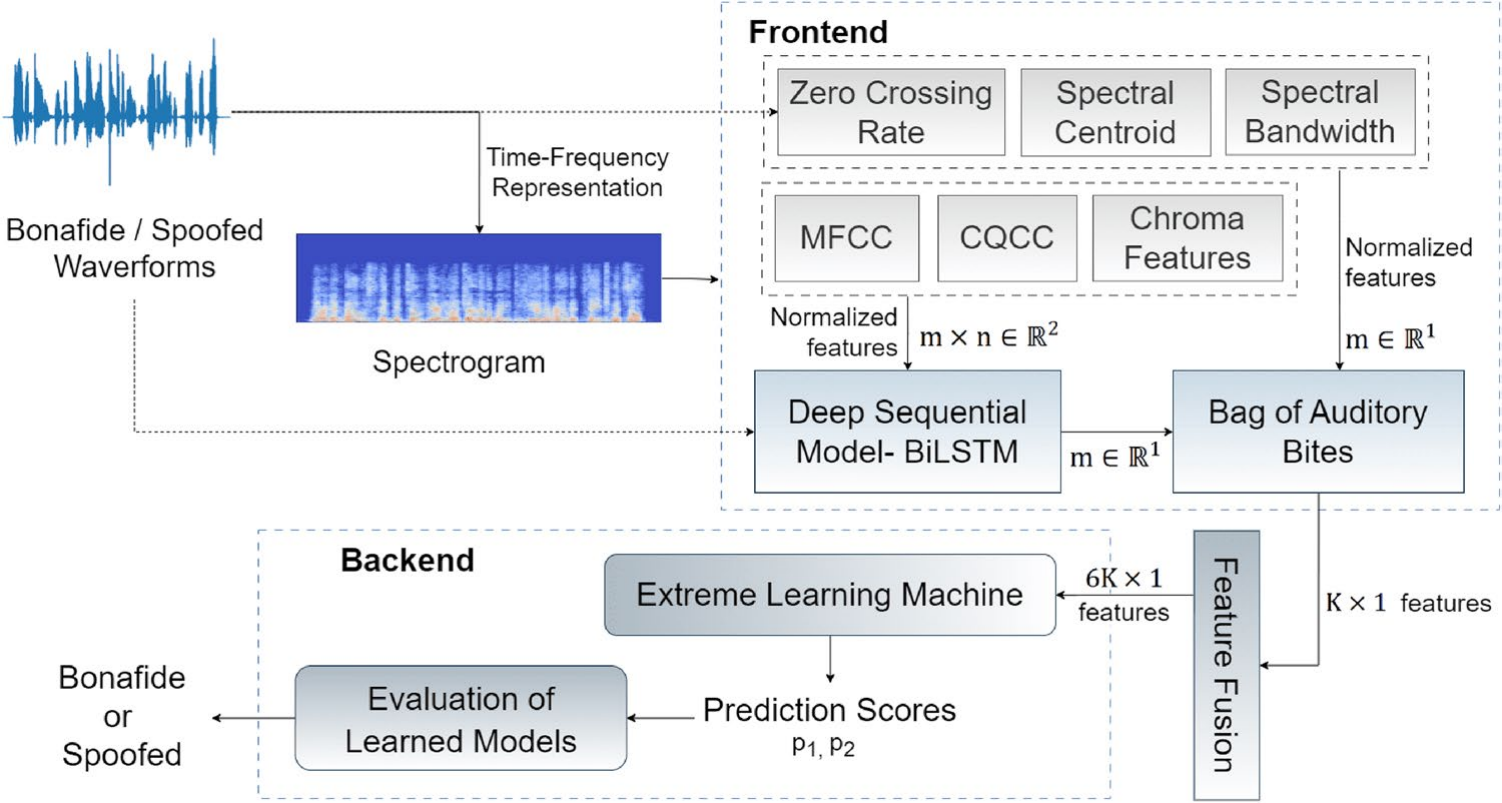
Overall architecture of the proposed Automated Spoof Detection System.

Figure 2 illustrates the overall framework of the proposed automated spoof detection system. We propose a novel technique for processing audio features of varied lengths *m*. The one-dimensional features $m\in R^1$ and two-dimensional features $m\times n \in R^2$ extracted from raw features and spectrograms are processed using the Bag of Auditory Bites (BoAB) algorithm. The BoAB model similar to the Bag of Words (BoW) in Natural Language Processing is created to balance the number of features for classification. The one-dimensional feature arrays of Zero Crossing Rate (ZCR), Spectral Centroids and Bandwidths of each audio sample are clustered into different auditory bins using the BoAB algorithm. The Mel-Frequency Cepstral Coefficient (MFCC), Constant-Q Cepstral Coefficient (CQCC) and Chromagram features are 2-dimensional. Hence, each time array is processed separately using a deep sequential learning Bi-LSTM model in a many-to-one scheme to predict a single value for the entire time-wise sequence^38,39^. The sequential model is trained to produce a one-dimensional array $m\in R^1$ of values (representing the raw signal strengths) corresponding to each time point. These are then clustered into several bags of auditory bites, the count of each of which is then considered as a separate fixed set of $K\times 1$ features for the entire audio sequence. The patterns in BoAB features of each characteristic are fused to $6K\times 1$ features and then learned by an Extreme Learning Machine (ELM) model that predicts the confidence of genuineness.

#### Feature extraction from speech signals

A strong description must be formulated to capture any distortion of cloning algorithm artefacts and the dynamic features of the vocal tract of a human speaker in real audio to build a powerful voice anti-spoofing system. The audio feature extraction needs to be done, which is a digital and analogue signal conversion process to eliminate any unwanted noise and balance out the time-frequency ranges. Spectrograms from the frequency domain extract features from the audio signals^40^. A spectrogram is a visual graph that displays how the frequencies of a signal change over time. It comprises two axes, one for time and one for frequency, with the intensity or colour of each point representing the amplitude of a frequency at a given time. It is generated by dividing the audio data into brief intervals and computing the Discrete Fourier Transform (DFT) for each segment, determining the magnitude of the frequency spectrum. These magnitude spectra, illustrating the amplitudes of various frequency components, are subsequently organized over time to construct the two-dimensional spectrogram. From this spectrogram, features relevant to identifying human vs. machine-generated tracts are extracted.

1 $$\begin{aligned} ZCR_n&= \sum _m|sign(s_m) - sign(s_{m-1})|win(n-m) \end{aligned}$$

2 $$\begin{aligned} SC_n&= \frac{\sum _m freq_{mn} s_m}{\sum _m s_m} \end{aligned}$$

3 $$\begin{aligned} SB_n&= \sqrt{\sum _m s_m(freq_{mn} - SC_n)^2} \end{aligned}$$

Zero Crossing Rate (ZCR) measures the frequency of sign changes in the signal, producing one ZCR value per frame, resulting in a feature vector with the same length as the number of frames. In Eq. 1, $ZCR_n$ measures the number of times a signal crosses the zero line during a given time frame *n* within window width $\text {win}$. The *signum* function *sign*() produces $-1$ if the signal strength $s_m$ is less than 0, and $+1$ if strength is more than 0. It is primarily employed for assessing human speech signals, effectively distinguishing speech from background noise, as speech usually has a higher ZCR than noise, and is a potent indicator to detect audio manipulations.

Spectral Centroid (SC) calculates the weighted mean of frequencies in each frame, resulting in a feature vector with one SC value per frame. Calculated using Eq. 2, it identifies unique voice characteristics and audio spoofing by measuring the weighted mean of frequencies. Here, $freq_{mn}$ is the magnitude of the Fourier transform at frame *n* and frequency bin *m*. Although there may be a spurious rise in the centroid at the start of the signal due to undefined silence in the audio sequence. It can impact the authentication process by leading to false positives or negatives, increasing feature variability, and complicating feature extraction. Techniques such as trimming the initial silence or applying threshold-based techniques to ignore low-energy segments may attenuate their effects.

Spectral Bandwidth (SB) assesses the spread of frequencies around the centroid, producing one SB value per frame, creating a feature vector of similar length. The bandwidth of a signal is the range of frequencies in which it oscillates. It is determined by calculating the average distances between the upper and lower frequencies in a continuous range with respect to the spectral centroid. The values in the range are weighted and averaged by the signal strength with respect to the spectral centroid $SC_n$ (Eq. 3). Analyzing spectral bandwidth helps identify discrepancies, modifications, or manipulations in the frequency spectrum, indicating audio spoof presence or background noise concealment.

**Figure 3:**
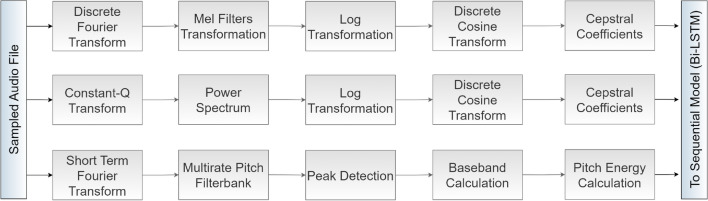
Flow of extracting Chromagrams, MFCCs, and CQCCs.

The Chromagram, MFCC, and CQCC are considered for extracting 2-dimensional features as shown in Fig. 3. Chroma-based features, also known as pitch class profiles, measure the frequency of sound, allowing them to be categorized by pitch and enabling users to interpret the tonal quality of the signal. Chromagram maps frequencies to 12 pitch classes, providing a 12-dimensional feature vector per frame, leading to a matrix with dimensions $12\times (number\; of\; frames)$, which is particularly useful for machine-generated and manipulated audio. Their static pitch differences are used for high-level semantic analysis.

Furthermore, the Mel-Frequency Cepstral Coefficients (MFCC) is the most commonly used feature coefficient in spoof detection. This approach involves comparing pitches on the Mel scale, designed to reflect how the human auditory system perceives sound. To calculate the MFCCs, FFT (Fast Fourier Transform) or DFT (Discrete Fourier Transform) is applied to the speech signal, which creates an audio spectrum. This spectrum is then filtered with triangular, gaussian, etc. filters to convert it to the Mel-scale. The spectrum is then taken through a logarithmic transformation and a Discrete Cosine Transform (DCT) to generate the MFCCs. MFCC typically results in 13 coefficients per frame, creating a matrix with dimensions $13\times (number \;of \;frames)$.

The Constant Q Transform (CQT) generates Constant-Q Cepstral Coefficients (CQCCs), which provide higher frequency resolution at lower frequencies and more temporal resolution at higher frequencies. The extraction of these features starts with the application of CQT, which transforms the time domain into the frequency domain, followed by taking the power of the spectrum and a logarithmic operation, uniform re-sampling to convert the geometrically spaced CQT bins to linearly spaced bins, and finally, the application of the DCT. They generally produce 13 coefficients per frame, forming a matrix with dimensions $13\times (number\ of\ frames)$. These features are extensively used for LA and PA spoof detection in ASV systems and perform better than other extracted features.

#### Significance learning from sequential model

Long Short-Term Memory (LSTM) neural architectures are sequential models for learning and forecasting time-series data of varied lengths^38^. The models capture long-term context-sensitive dependencies. Bidirectional LSTMs (Bi-LSTM) utilise both past and future context to predict the next value in a sequence accurately. This model can learn the sequence patterns and use this knowledge to make reliable predictions. We used a Bi-LSTM to take in the cepstral coefficients at each timestamp t and predict the signal strength at the next timestamp $t+1$. Signal strengths refer to the amplitude of the sampled digitised audio sequence. The model is trained with input-output training pairs constituting the cepstral coefficients at each time point t throughout the dataset and their corresponding raw signal strengths. The sequential model is designed with a layer of embedding layer that extrapolates the input signal strength to a vector of length 128, further attaching the embedded vector through a Bidirectional LSTM of 64 neurons. The model is regularized with a dropout of 50%. The network was trained to predict single values for every set of coefficients over each time point with a *sigmoid* activation function, signifying the relevance of the coefficients. The significance of predictions is directly related to their accuracy, with more accurate predictions holding higher significance and less accurate predictions indicating lesser significance. To maintain the integrity of both classes, the network was trained exclusively on the bonafide class, enabling us to assess the reproducibility of both classes. Additionally, this process facilitated the reduction of dimensionality from two dimensions to a single array of values, which represents the reproduced signal. Through this sequential model, new values are generated for each amplitude of the original signal, with a closer resemblance indicating more significant cepstral coefficients and a greater disparity indicating less significant coefficients.

#### Bag of Auditory Bites (BoAB)

Machine learning algorithms require feature attributes to be of the same dimension. Due to the varying lengths of audio signals, the extracted features are also varied. The BoAB technique was designed to regularise the number of features to a defined set of values. It is wrapped around K-Means algorithm, which compresses and bounds dimensionality to a finite count. BoAB employs the train set to acquire a vocabulary of auditory words, which are then transformed into codewords. Algorithm 1 describes the two-stage procedure of BoAB to regularise the number of features.

**Algorithm 1:**
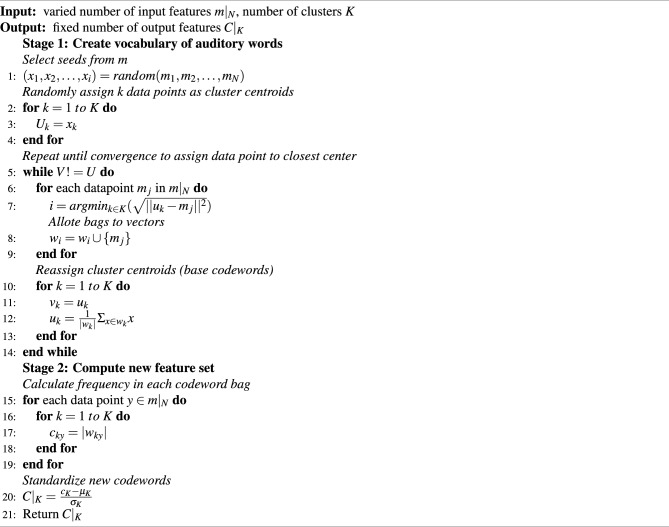
Bag of Auditory Bites

Firstly, the dictionary is created using k-means clustering to group similar auditory bites into k distinct sets of clusters, with cluster centroids $u_k$ representing the base codewords. Initially, *k* random datapoints are considered as the cluster centroids (codewords). We further compute the euclidean distances between each datapoint $m_j$ and the cluster centroids $u_k$, and assign the $i^{th}$ bag to the $j^{th}$ vector based on minimal distance. Next, new centroids are calculated as the mean sum of all datapoints assigned to the cluster $w_k$. *U* and *V* represents the cluster centroids of adjacent iterations. The procedure is repeated until the cluster centroids *U* and *V* are equal. These *k* codewords in the dictionary are now the new feature set. The second step is to create feature values for each training waveform by making histograms of the *k* codewords. $c_{ky}$ is the number of features of the $y^{th}$ datapoint clustered into the $k^{th}$ bag, while $c_K$ is the number of features of all datapoints in the dataset clustered into the entire *K* bins. For optimal performance, the data points are further standardized to $C|_K$ by subtracting the mean from each data point. Then, each data point is divided by the standard deviation, resulting in a mean of 0 and a standard deviation of 1. The regularity of the auditory bites in a timestamp denotes the feature values of the k-numbered feature set. The value of *k* is altered from 100 to 300 and tested with different machine learning algorithms.

#### Learning models and evaluation

Extreme learning machines (ELMs)^41^ represent a class of artificial neural networks known for their high performance and robustness in classification tasks. Unlike the traditional iterative training procedure, ELMs accelerate training time by updating network weights only once during the learning process. We trained and tested the BoAB features on Extreme Learning Machine (ELM) to detect whether the incoming features were genuine or spoofed. ELMs are powerful tools for classification due to their non-linear mapping abilities and quick training times with strong generalization capabilities. They constitute a single hidden layer that captures the input data patterns simultaneously. ELMs are universal approximators with the ability to accurately mimic any continuous function, especially when they have a large number of hidden neurons in a single-layer hidden structure. This study designed ELM with 1000 hidden neurons to maintain parity and sufficiency with the number of input features. The two output neurons were coupled to the softmax activation function, which forecasts probability scores indicating the degree of confidence with which the input stream belongs to either class. Additionally, various settings for k Nearest Neighbour (kNN), Support Vector Machine (SVM), and Random Forest (RF) were tested for comparison.

**Figure 4:**
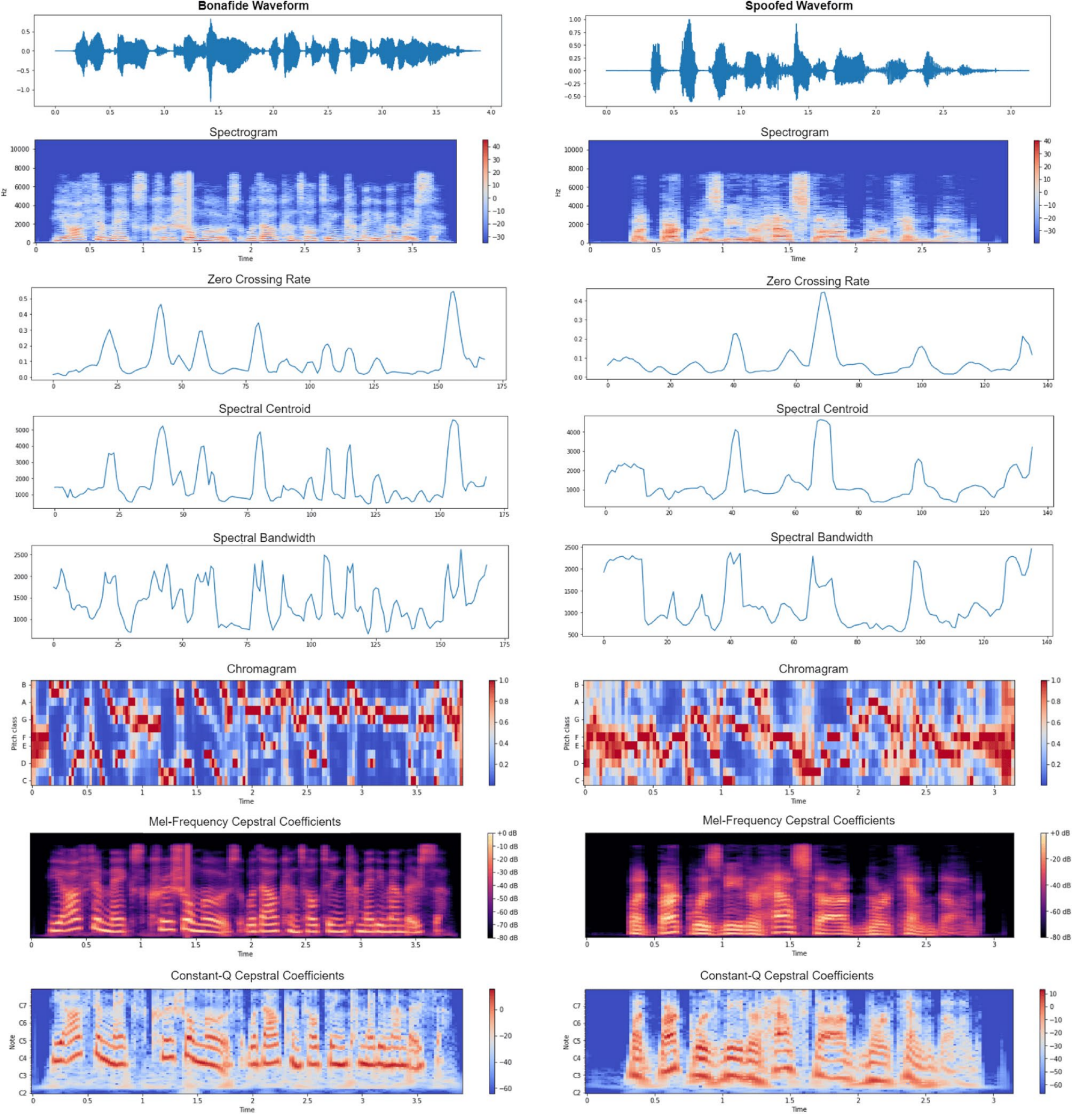
Waveforms and the several descriptors extracted from the bonafide and spoofed audio of speaker LA_0046.

All models were evaluated in terms of accuracy, precision, and recall computed from the confusion matrices. Each row of the confusion matrix corresponds to the number of an actual class, and the columns correspond to the number of predicted classes. The accuracy of a model is the frequency with which it rightly categorises a sample to its representative class^42^. Precision is the proportion of accurately recognised positive outcomes compared to all projected positive outcomes. In contrast, recall relates to the ratio of accurately identified good outcomes to all real positive outcomes. The accuracy of a biometric system is measured using Equal Error Rate (EER). It is the rate at which incorrect results (both False Positives and False Negatives) occur equally and is determined by plotting the False Negative Rate (FNR) and False Positive Rate (FPR) on the same graph. The point of error at which the two curves intersect is the EER, expressed as a percentage. A lower percentage denotes a more precise system.

## Results and discussion

This study aims to apply machine learning to classify genuine and spoofed speeches by combining audio file features extracted from the raw audio, and time-frequency converted spectrograms, which are then reduced to a lower dimension space using sequential models. Standardization of the feature set of various sizes to fixed dimensions is done through Bags of Auditory Bites (BoAB) clustering. The performance of the proposed architecture was tested on the Logical Access attack and Deepfake attack suites of the ASVspoof2021 challenge dataset. The datasets are split into the train and test parts in the ratio of 80:20.

Figure 4 displays the spectrogram of the raw bonafide signal $LA\_E\_1717780$ and the logical access spoofed waveform $LA\_E\_4237738$ of $Speaker\_ID$ $LA\_0046$ in the frequency domain. It also shows their zero crossing rates, spectral centroid, spectral bandwidth, and the two-dimensional MFCCs, CQCCs, and chromatograms. It is evident that there are very slight differences between the waveforms and their characteristics. Generally, it is observed that pitch classes are highly distributed in spoofed signals, while cepstral coefficients are more refined in bonafide waveforms.

**Figure 5:**
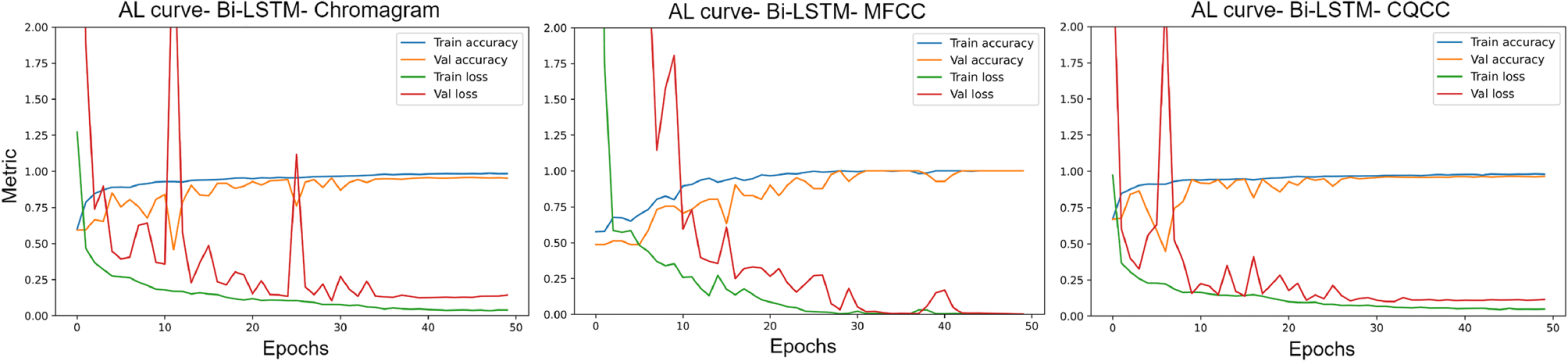
Accuracy and Loss curves corresponding to the training of Bi-LSTM on Chromagram, MFCC and CQCC timestamps.

**Figure 6:**
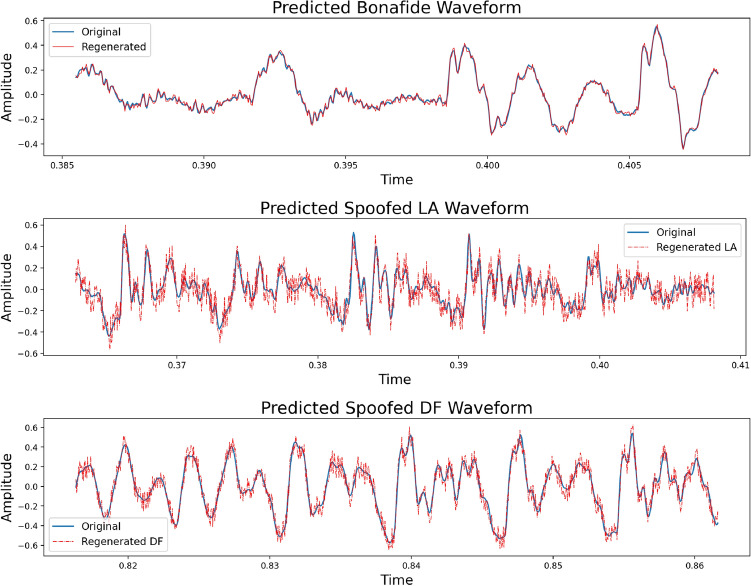
Predicted waveforms by the Sequential Bi-LSTM model.

The cepstral coefficients and chroma features of the bonafide class at each time stamp were used to train the Bidirectional LSTM model to predict single amplitude values across time. The model was optimized with the *Adam* optimizer configured with the default settings and the *CrossEntropy* cost function. The train and validation curves of the Bi-LSTM model on chromagram, MFCC, and CQCC separately are illustrated in Fig. 5. The declining loss curves depict the increasing capability of the sequential model to predict the next signal amplitude in the waveform. However, the loss curves fluctuate in the initial epochs and are smooth towards the end, as they have maximally learned audio bits generated from human tracts to a great extent. By training the model only on the genuine class, the ability of model to predict the next snippet in artificially synthesized audio waveforms becomes challenging. The reproduced bonafide and spoofed signals were generated by the sequential model using a minute sampled portion of cepstral coefficients, as shown in Fig. 6. The predictions of the model exhibited noticeable fluctuations, particularly in the case of the regenerated spoofed LA and DF waveforms. The differences were more pronounced in the regenerated LA signals. Additionally, a closer look at the original spoofed signal shows a smoother waveform compared to the bonafide signal (Tables 1, 2).

**Table 1:** Performance of Machine Learning algorithms on K = 100 cluster codes of BoAB on LA dataset.

Model	Accuracy (%)	Precision (%)	Recall (%)	EER (%)
kNN, k = 3	86.32	87.67	86.51	17.33
SVM, linear kernel	83.75	82.46	83.67	25.28
RF, depth = 20	84.87	81.62	83.37	25.65
ELM, hidden = 500	85.07	84.33	85.58	22.52
ELM, hidden = 1000	85.10	85.03	85.12	20.48
ELM, hidden = 1500	**87.95**	**85.83**	**87.44**	**16.90**

Significant values are in bold.

**Table 2:** Performance of Machine Learning algorithms on K = 100 cluster codes of BoAB on DF dataset.

Model	Accuracy (%)	Precision (%)	Recall (%)	EER (%)
kNN, k = 3	80.56	82.77	78.05	19.34
SVM, linear kernel	86.61	86.38	85.14	14.64
RF, depth = 20	86.90	81.62	83.37	14.97
ELM, hidden = 500	89.33	84.33	85.58	10.44
ELM, hidden = 1000	90.39	91.10	89.82	6.12
ELM, hidden = 1500	**92.15**	**92.52**	**90.56**	**4.49**

Significant values are in bold.

BoAB features for the audio descriptors of the LA and DF train set were computed separately based on Algorithm 1. Extracted features from the test audio files were then clustered based on the learned cluster centroids of the train set. The frequencies of each bag of auditory bites are considered the new fixed feature set. The features were further fit and tested with the kNN, SVM, RF, and ELM machine learning algorithms. Table 1 and Table 2 show the evaluation metrics of the performance of the classifiers on different settings for K = 100 number of cluster codes considered for fixating features on the two data parts. K = 100 would denote 100 new codewords for each feature representing ZCR, spectral centroid, and the others. Thus, the final feature set would account for 6*100  =  600 new feature values when K = 100.

A neighbourhood of k = 3 was considered for the k nearest neighbour algorithm, and the linear support vector classifier was trained as tested. In contrast, a depth of 20 levels was considered for the random forest ensemble classifier. However, none of the settings could capture the intrinsic patterns embedded in the signal features. The neurons in the ELM hidden layer were varied from 500 to 1500 to find the optimal and best-converging set. We observed that increasing the hidden neurons captured a more expansive data distribution space, leading to better prediction performance. Hence, a sufficient number of hidden neurons compared to the number of features ensured convergence and a minimal error rate.

A second set of experiments were performed by varying the number of codewords from 100 to 300 to train the ELM with 1500 hidden neurons (Table 3 and Table 4). A decline in performance was observed when the number of codeword features out-numbered the number of hidden neurons and vice versa. A code set of 1200 features performed best for both data parts compared to their lower and higher counterparts. The computational time increased exponentially with the increase in codewords K due to the more significant number of Euclidean distance calculations involved in the clustering process. The best-performing models detected spoofs at an accuracy of 95.04% in LA detection and 96.43% in DF detection.

**Table 3:** Performance of Extreme Learning Machine with 1500 hidden neurons on varied cluster codes per descriptor for LA track.

K (codewords/feature)	Total feature set	Accuracy (%)	Precision (%)	Recall (%)	EER (%)
100	600	87.36	86.57	87.89	16.27
150	900	92.51	90.74	91.36	14.43
**200**	**1200**	**95.04**	**94.49**	**95.67**	**13.78**
250	1500	90.49	89.32	90.18	13.95
300	1800	87.43	87.18	88.26	16.61

Significant values are in bold.

**Table 4:** Performance of Extreme Learning Machine with 1500 hidden neurons on varied cluster codes per descriptor for DF track.

K (codewords/feature)	Total feature set	Accuracy (%)	Precision (%)	Recall (%)	EER (%)
100	600	93.89	93.26	93.97	2.90
150	900	94.14	95.73	93.54	2.42
**200**	**1200**	**96.43**	**97.42**	**95.39**	**1.61**
250	1500	92.97	93.84	92.02	3.76
300	1800	92.93	92.96	93.21	3.85

Significant values are in bold.

**Figure 7:**
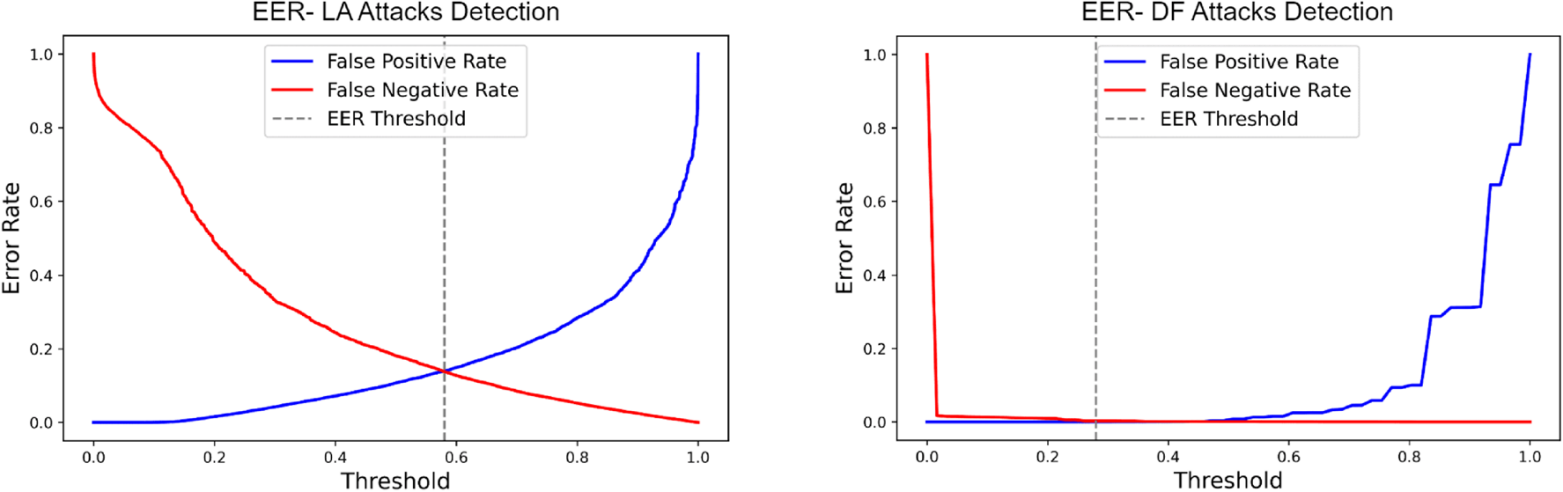
Equal error rate plots of extreme learning machine in the optimal settings for LA and DF tasks.

The EER of ELM in the optimal setting for the two experiments on the test data is shown in Fig. 7, with the false acceptance and rejection rates intersecting at 13.78% with a threshold of 57.00%. Meanwhile, in deepfake attack detection, the EER is notably low at 1.61% with a threshold of 28.00%.

**Figure 8:**
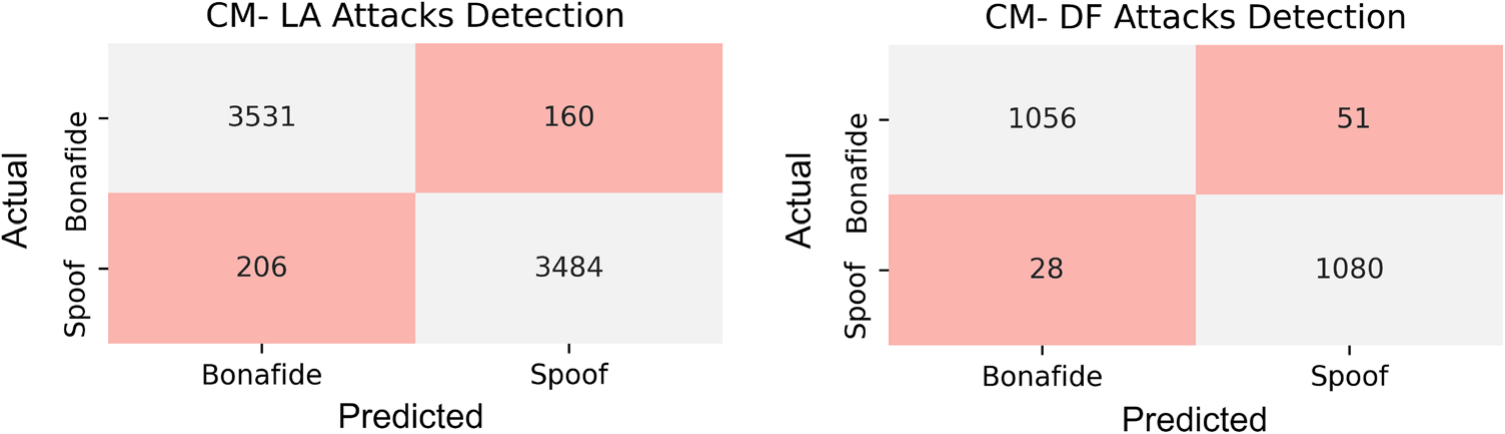
Confusion matrices plotted for the ELM trained and tested to detect logical and deepfake attacks.

The confusion matrices corresponding to the optimal ELM for the test sets of the two data parts are presented in Fig. 8. While the models seem to have performed consistently on different attack types, a slight performance boost is observed on the DeepFake spoof detection. This approach detected almost 98% of synthesized content from the test dataset. The lack of speaker authentication during the deepfake generation process has left clear signatures detected using feature extraction methods.

The model had been further experimented on the evaluation split of the ASVSpoof dataset tracks (Tables 5 and 6). For both the evaluation sets of LA and DF tracks, ELM with 1500 hidden neurons achieves the best performance with 200 codewords/feature. It yields the highest accuracy, precision, and recall, along with the lowest EER. Increasing the codewords or features to 250 results in a notable performance drop across all metrics for both tracks. This indicates that adding more codewords beyond a certain point does not necessarily improve the model effectiveness. At 150 codewords, performance is lower than at 200 but still relatively high, suggesting that a moderate number of codewords is beneficial. Overall, both tracks illustrate that 200 codewords consistently provide the best balance between model complexity and performance, with the DF track showing lower EER values, indicating greater adaptiveness against false acceptance/rejection.

**Table 5:** Performance of Extreme Learning Machine with 1500 hidden neurons on varied cluster codes per descriptor for the evaluation set of the LA track.

K (codewords/feature)	Total feature set	Accuracy (%)	Precision (%)	Recall (%)	EER (%)
150	900	91.89	91.12	90.54	14.72
**200**	**1200**	**94.67**	**95.11**	**94.29**	**12.22**
250	1500	90.15	90.56	90.78	13.01

Significant values are in bold.

**Table 6:** Performance of Extreme Learning Machine with 1500 hidden neurons on varied cluster codes per descriptor for the evaluation set of the DF track.

K (codewords/feature)	Total feature set	Accuracy (%)	Precision (%)	Recall (%)	EER (%)
150	900	93.03	93.56	94.47	2.11
**200**	**1200**	**95.21**	**95.33**	**95.22**	**1.18**
250	1500	94.82	93.68	94.88	2.21

Significant values are in bold.

These results demonstrate that the performance of ELM remains consistent and effective across the evaluation sets for both the LA and DF tracks of the ASVSpoof dataset, with optimal results observed at K = 200 as observed with the test set split of the dataset.

### Discussion

Our novel methodology integrates a multitude of auditory artifacts relevant to constructing an anti-spoofing system. It explores the temporal and spectral properties of genuine and spoofed recordings in the front end and utilizes simple machine learning algorithms in the back end. It is important to compare our proposed feature descriptor for voice spoofing detection with the existing baseline features in order to evaluate the effectiveness of our integrated feature set. We have compared our model against baseline feature extraction methods such as MFCC, LFCC, and wav2vec outlined in Table 7.

**Table 7:** Performance comparison with related works on ASVspoof2021 challenge.

References	Approach	Results (EER)
Caceres et al.^25^	MFCC + Lightweight TDNN	LA-7.51
Arif et al.^33^	ELTP + LFCC + DBi-LSTM	LA-0.74
Chen et al.^32^	ResNet-TDNN + one class learning	LA-5.46 DF-20.33
Luo et al.^35^	Capsule Network	LA-3.19
Wang et al.^30^	Wav2vec + LFCC + Bi-LSTM	LA-7.35 DF-6.10
Zhan et al.^26^	WCQCC + AZCR	LA-3.72
Chakravarty et al.^29^	MFCC + GTCC + LSTM	DF-5.10
Dişken et al.^36^	SE-ResNet	LA-10.99 DF-24.39
Xie et al.^34^	TDL	DF-11.23
Chen et al.^37^	CQT + DCN	LA-4.83
Proposed	Temporal + Spectral features + Bi-LSTM + BoAB + ELM	LA-12.22 DF-1.18

Data imbalance is a significant concern previous researchers have yet to contemplate, which could account for the very high equal error rates. Authors have mostly adapted to using a single audio descriptor, specifically from spectrograms as in^25^ and^33^, which could prove insufficient as the traces of spoofing are very sparse. Different variants of deep neural networks have been extensively researched in the latest ASVspoof challenges. Deep Bidirectional LSTM and Time Delay Neural Networks have been designed specifically to process and find patterns temporally across the entire sequence of audio^30,32^. However, their implementation as a classifier has increased the complexity of training and testing feature values, yielding just fine results as projected by Wang et al.^30^. Zhang et al.^26^ demonstrated that incorporating a segment-based word CQCC and average ZCR technique led to a substantial reduction in Equal Error Rate (EER) when compared to other state-of-the-art methods. Learning of the bonafide class was proposed by Chen et al.^32^, in which the positive class was well characterized by training data instances, identifying any outlier as spoofed content. We utilized a similar logic in learning the best representations of the bonafide class through the significance learning of Bi-LSTM module. The deep correlation network (DCN) significantly improved detection performance by learning and utilizing common information between different audio features^37^. The proposed light-weight method exhibits improvements over state-of-the-art approaches on the ASVspoof2021 dataset, achieving a whopping improvement rate of 95.16% on Equal Error Rate (EER) for the most infrequent and recent DeepFake (DF) attacks.

**Figure 9:**
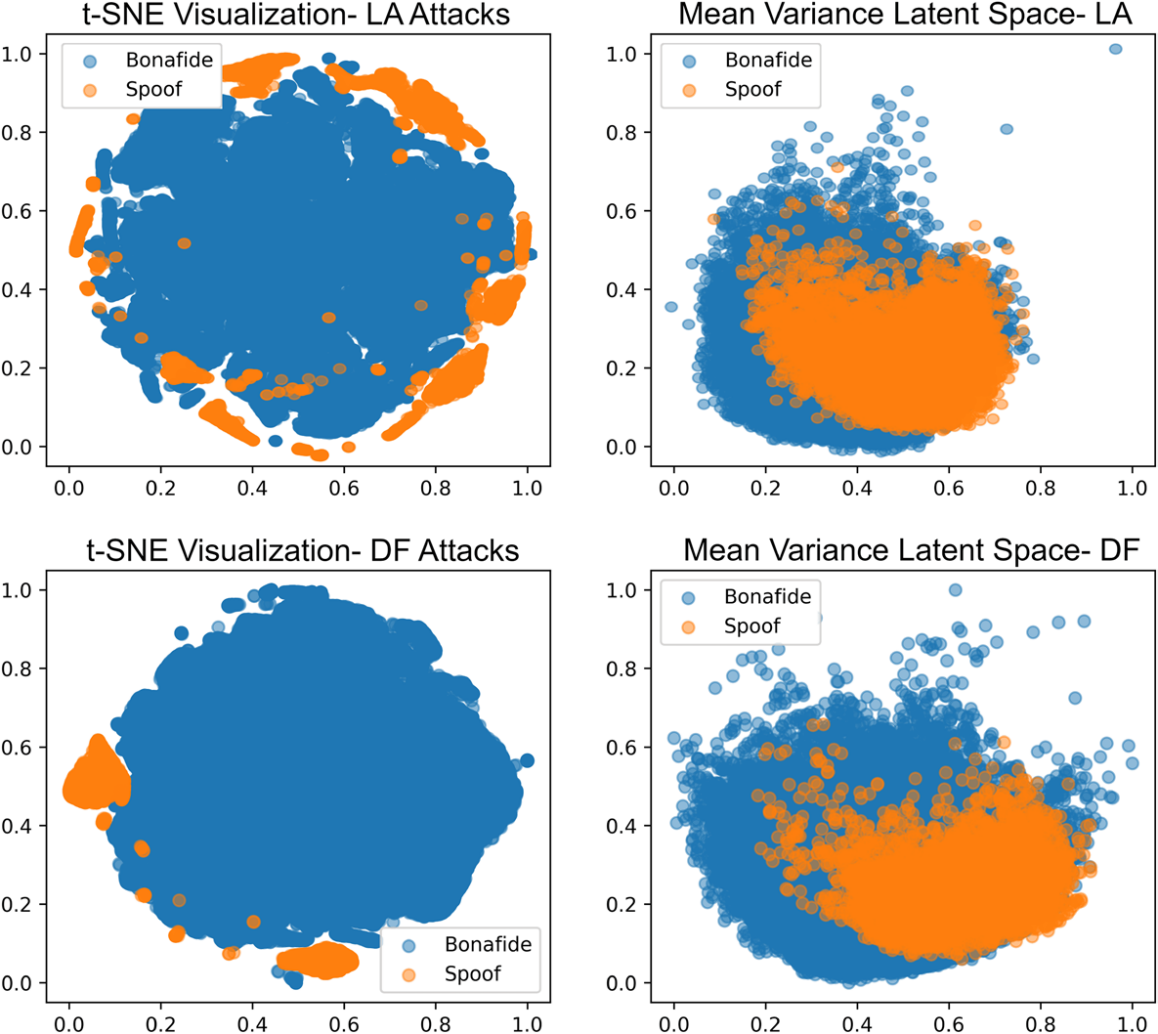
t-SNE and mean-variance visualizations of converged BoAB features in LA and DF datasets.

We recognize the significance of a comprehensive comparison between the latent space generated by the t-SNE clustering process^43^ and that produced by the mean-variance approach^44^. Figure 9 illustrates the t-SNE and mean-variance visualizations of both datasets. T-SNE is a potent dimensionality reduction method excelling at visually capturing intricate structures within high-dimensional datasets, making it invaluable for discerning clusters and patterns. Conversely, mean-variance visualization offers valuable insights into the distribution characteristics by revealing the spread and concentration of data points. The structural representations projects spoofed data beyond the boundaries of the bonafide signal spread in the t-SNE visualizations for both datasets when compared with the mean-variance visualizations. This indicates the capture of variational features from both datasets. Notably, specific features in the data point projection from the spoofed dataset are visually accumulated, leading to the convergence of ASVSpoof detection.

Results show that our methodology outperforms existing approaches by finding a representation that signifies the relevance of each timestamp in two steps. First, by trying to predict the amplitude of the following timestamp and, secondly, by clustering the time-width feature vector into bags of auditory bites. Nevertheless, the model could be easily fine-tuned to more diverse audio samples from similar attack scenarios or even an entirely different attack category, such as a physical access attack.

The computational complexity of the sequential Bi-LSTM and ELM can be demanding, potentially limiting real-time applicability and scalability. The interpretability of the proposed model is also limited, making it difficult to understand the decision-making process fully. While the study focuses on detecting fully spoofed audio inputs, there is a significant gap in identifying partially spoofed audio, which can be critical in more complex attack scenarios. Future work could incorporate explainable AI (XAI) to enhance model transparency, aiding user trust and debugging. Developing methods to identify and analyze manipulated audio segments, integrating granular feature extraction and hybrid models, would significantly improve effectiveness in complex attack scenarios.

## Conclusion

The study proposes to integrate audio file components taken from raw audio files and time-frequency representation spectrograms that are compressed to a lower-dimension space using sequential prediction models. The feature set of various sizes is then standardized by the Bags of Auditory Bites (BoAB) feature standardizing technique. After the feature space has been transformed, a machine learning algorithm is applied to map it to predictions that differentiate between genuine and spoofed speeches. The framework is trained and tested on the logical access attacks and deepfake attacks of the ASVspoof challenge validating its efficacy. Experiment findings showed favorable results on synthesized deepfake attacks. Nevertheless, we discovered that logical access attacks are more challenging to detect since voice conversion algorithms take voice samples as input rather than deepfakes generated from prompts. Lastly, several methods are available to determine if an audio input is fully spoofed; not many methods can identify spoofed audio in a specific portion of the input. Future work aims to enhance the computational efficiency and interpretability of the ASV detection systems, focusing on identifying both fully and partially spoofed audio for improved efficacy against complex attack scenarios.

## Data Availability

Source code of the framework and the dataset are made available at http://www.mirworks.in/downloads.php.
